# A Broad-Spectrum Chemokine Inhibitor Drives M2 Macrophage Polarization Through Modulation of the Myometrial Secretome

**DOI:** 10.3390/cells14070514

**Published:** 2025-03-30

**Authors:** Adam Boros-Rausch, Anna Dorogin, Lubna Nadeem, Oksana Shynlova, Stephen James Lye

**Affiliations:** 1 Lunenfeld Tanenbaum Research Institute, Mount Sinai Hospital, Toronto, ON M5G 1X5, Canada; aboros@lunenfeld.ca (A.B.-R.); dorogin@lunenfeld.ca (A.D.); nadeem@lunenfeld.ca (L.N.); lye@lunenfeld.ca (S.J.L.); 2 Department of Physiology, University of Toronto, Toronto, ON M5S 1A1, Canada; 3 Department of Obstetrics & Gynecology, University of Toronto, Toronto, ON M5S 1A1, Canada

**Keywords:** uterus, myometrium, pregnancy, labor, mechanical stretch, broad spectrum chemokine inhibitor, macrophage, cytokine, inflammation, mass-spectrometry

## Abstract

The uterine smooth muscle (myometrium) is an immunomodulatory tissue capable of secreting multiple chemokines during pregnancy. We propose that before term labor, chemokines secreted as a result of mechanical stretch of the uterine walls by the growing fetus(es) induce infiltration of maternal monocytes into myometrium, drive their differentiation into macrophages, and induce pro-inflammatory (M1) polarization, leading to labor contractions. This study used high-throughput proteomic mass-spectrometry to investigate the underlying mechanisms and explored the therapeutic potential of a broad-spectrum chemokine inhibitor (BSCI, FX125L) in modulating these effects. Primary myocytes isolated from the myometrium of term pregnant women were subjected in vitro to static mechanical stretch. Proteomic analysis of stretched myocyte-conditioned media (CM) identified significant upregulation of chemokine-related pathways and ECM degradation proteins. CM induced in vitro differentiation of human monocytes to macrophages and polarization into an M1-like phenotype characterized by elevated ROS production. BSCI treatment altered the myocyte secretome, increasing tissue-remodeling and anti-inflammatory proteins, Annexin A1 and TGF-β. BSCI-treated myocyte secretions induced Annexin A1 expression in macrophages and enhanced their phagocytic activity. We conclude that factors secreted by mechanically stretched myocytes induce pro-inflammatory M1 macrophage polarization, while BSCI modulates myocyte secretome, which reprograms macrophages to a homeostatic M2-like phenotype, thus reducing inflammation. When treated with BSCI, M2-polarized macrophages reduced myocyte-driven collagen gel contraction, whereas M1 macrophages enhanced it. This study reveals novel insights into the myocyte–macrophage interaction and identifies BSCI as a promising drug to modulate myometrial activity. We suggest that uterine macrophages may represent a therapeutic target for preventing preterm labor in women.

## 1. Introduction

Preterm birth (PTB), delivery before 37 weeks of gestation in women, is the leading cause of newborn morbidity and mortality globally [1]. Premature babies have increased risks for health problems throughout their lives, placing a heavy burden on families and healthcare systems [2]. PTB affects a significant portion of pregnancies, with 10.4% of births in the US alone classified as preterm in 2022 [3]. Approximately 25–30% of PTBs are iatrogenic (medically indicated), based on complications of the pregnant patient, such as placental deficiency or fetal anomalies [4]. The remaining 70–75% result from idiopathic spontaneous PTB (sPTB) or preterm premature rupture of membranes (PPROM), caused by infection or inflammation [5]. Understanding the mechanisms behind these pregnancy complications is crucial for developing therapies to prevent PTB.

It is now well established that inflammation is present in the physiological term labor processes [6]. We and others have shown that the uterine smooth muscle (myometrium) is an immunomodulatory tissue capable of secreting multiple cytokines and chemokines during labor [7,8,9,10]. It has been demonstrated that this secretion is induced during late gestation by the mechanical stretch of the uterine walls by growing fetuses [10,11,12]. The increased secretion of cytokines and chemokines leads to the infiltration of leukocytes from maternal circulation into the uterine tissues, further propagating inflammation to aid in labor onset. This phenomenon is seen in singleton-term pregnancies and is exaggerated by uterine distension in multiple pregnancies (i.e., twins, triplets), disproportionately contributing to PTB rates [13].

Among the leukocytes infiltrating the myometrium, monocytes (later differentiating into tissue macrophages) are essential throughout pregnancy and potentially contribute to the labor process [14,15,16,17,18]. Macrophages exhibiting a phenotype, known as M1, are characterized by the production of pro-inflammatory cytokines interleukin (IL)-1β, tumor necrosis factor (TNF)-α, and reactive oxygen species (ROS) [19]. In contrast, M2 macrophages exhibit a homeostatic phenotype characterized by the production of anti-inflammatory cytokines IL-10 and transforming growth factor (TGF)-β [19]. Our previous rodent studies revealed that macrophages populate the uterine muscle layer throughout pregnancy, increasing at late gestation as well as during both term and PTL [7,9,16,17]. It has been shown that macrophage depletion in a rodent model prevents infection-induced PTB and cervical remodeling [20,21,22]. Further evidence comes from murine studies, showing beneficial effects of the adoptive transfer of homeostatic M2 macrophages capable of preventing PTB and improving the newborn health of pups, in a model of in utero sterile inflammation induced by the intra-amniotic injection of the alarmin HMGB1 [23,24]. More recently, analysis of human and mouse uterine tissues reveals a dominance of M2 macrophages during late gestation that decreases during labor, supporting their role in maintaining uterine quiescence [23,24,25,26]. Although the exact link between macrophages and labor onset remains unclear, research suggests a potential direct or indirect macrophage–myocyte interaction [27,28]. Understanding how the myometrial tissue shapes the dynamic changes in macrophage phenotype in preparation for labor is essential. Our previous research indicates that direct contact and the possible exchange of materials exists between myometrial macrophages and myocytes, which promotes an increase in the expression of myocyte contraction-associated proteins (CAPs) involved in uterine contractions [29,30].

Managing PTB clinically often involves targeting myometrial contractility to delay labor onset. Anti-inflammatory prostaglandin inhibitors, such as NSAIDs, are common relaxants that suppress uterine contractions by reducing PG synthesis, thereby prolonging pregnancy [31]. In contrast, oxytocin and prostaglandins reinforce contractions and are used to induce labor, highlighting the opposing mechanisms that regulate myometrial activity [32]. Our previous studies have established the efficacy of a novel anti-inflammatory pharmaceutical agent, broad-spectrum chemokine inhibitors (BSCIs), in preventing uterine inflammation. To date, we have shown, (1) in vivo administration of the BSCI compound “BN83470” prevents infection (LPS)-induced PTB in mice by reducing uterine secreted cytokines and immune cell influx, without affecting fetal viability or weight [33]. (2) The BSCI compound “FX125L” successfully reduced inflammation in a non-human primate (NHP) PTB model caused by Group B Streptococcus infection, preventing uterine contractions and suppressing fetal cytokine secretion [34]. (3) In vitro, the BSCI inhibited monocytic cell migration across human endothelium [10] and (4) prevented human leukocyte migration in response to labor-associated cytokines [35]. (5) Our mechanistic studies indicated that FX125L inhibited intracellular signaling pathways in human myocytes, including activation of transcription factor NF-κB and chemokine secretion, which disrupted macrophage–myocyte interactions and in vitro myocyte contraction [29]. However, it remains unclear whether the BSCI affects the polarization of macrophages within the myometrium and the resulting implications.

We hypothesize that under physiological conditions, myometrial stretch influences the secretion of multiple proteins capable of polarizing macrophages to pro-inflammatory M1 phenotype, aiding in labor onset. We further hypothesize that the BSCI targets uterine myocytes, altering their secretion, decreasing myocyte contractility, and changing a macrophage to a homeostatic M2-like phenotype. Therefore, the objectives of this study were (1) to determine the impact of static mechanical stretch on the proteins secreted by myometrial cells (myometrial secretome) and the role of this secretome on monocyte differentiation to macrophages and their polarization to M1/M2 phenotypes; (2) to investigate whether the BSCI (FX125L) can change the secretion of uterine myocytes to favor an M2 versus M1 macrophage polarization; and (3) examine if BSCI-treated macrophages can influence the contractility of human myocytes. Primary smooth muscle cells isolated from term human myometrium and monocytes isolated from the peripheral blood of pregnant women were used in this study. We examined how myometrial stretch is linked to macrophage phenotypic modulation during labor onset and how the BSCI can modulate this system for PTB prevention in humans.

## 2. Materials and Methods

### 2.1. Ethics

All sample collections were approved by the Mount Sinai Hospital Research Ethics Board (MSH REB #04-0024E and #12-0007-E). Informed consent was obtained from all subjects involved in the study. Myometrial biopsies were collected from healthy term pregnant women undergoing an elective cesarean section after receiving written consent. Peripheral blood was collected from third trimester pregnant women (37–40 weeks’ gestation) into EDTA vacutainer tubes.

### 2.2. Myometrial Cell Isolation and Culture

Myometrial biopsies (N = 4) were collected in 50 mL falcon tubes containing 25 mL of ice-cold HBSS with Ca^2+^ and Mg^2+^ (HBSS+/+) supplemented with 2.5% HEPES (Wisent, Montreal, QC, Canada) and 1% Penicillin/Streptomycin (Pen/Strep, Lonza, Basel, Switzerland). Protocol for isolation of myometrial smooth muscle cells is described in Srikhajon et al. [9]. Briefly, myometrial tissues were washed in HBSS+/+ to remove blood, cut into small pieces (approximately 1 mm^3^) using small scissors, and blood vessels were carefully removed. Next, tissues were washed twice in HBSS without Ca^2+^ and Mg^2+^ (HBSS−/−), supplemented with 2.5% HEPES (Wisent, Montreal, QC, Canada) and 1% Pen/Strep (Lonza, Basel, BS, Switzerland), transferred to enzymatic digestion solution containing HBSS−/−, with 10% fetal bovine serum (FBS) (Wisent, Montreal, QC, Canada), 1 mg/mL collagenase 1A (Sigma-Aldrich, Oakville, ON, Canada), 1 mg/mL bovine serum albumin (BSA) (Wisent, Montreal, QC, Canada), 0.15 mg/mL DNase 1 (Sigma-Aldrich, Oakville, ON, Canada), and 0.1 mg/mL Trypsin inhibitor (Sigma-Aldrich, Oakville, ON, Canada), and placed in the rocking water bath at 37 °C. Following 1 h incubation, the digested myometrial tissue was pipetted 30 times and passed through a 70-micron filter to collect a single cell suspension in ice-cold dissociation solution (HBSS−/−, with 10% FBS (Wisent, Montreal, QC, Canada) and 1 mg/mL BSA (Wisent, Montreal, QC, Canada) to stop the enzymatic reaction. Undigested tissue was transferred to fresh enzymatic digestion solution for 1 h in the rocking water bath at 37 °C. Two cell suspensions were combined after the second round of digestion, centrifuged at 200× *g* for 10 min, washed with Dulbecco Modified Media (DMEM, Invitrogen, Carlsbad, CA, USA) containing 20% FBS (Wisent, Montreal, QC, Canada) + 1% Pen/Strep (Lonza, Basel, Switzerland), and passed through a 23 G ¾ gauge needle. The cells were seeded in a 10 cm tissue culture plate (Eppendorf, Mississauga, ON, Canada) or Flexcell plates (Flexcell International Corp., Burlington, NC, USA). The cells were grown in an incubator at 37 °C in 20% oxygen.

### 2.3. Vacuum-Driven Static Stretch of Myocytes

The impact of mechanical stretch on human myometrial cells was investigated in vitro using a vacuum-driven Flexcell computer system (FX-5000; Flexcell International Corp., Hillsborough, NC, USA) as described earlier [10]. Briefly, freshly isolated cells are plated on collagen-coated 6-well Flexcell plates at 300,000 cells/well density and grow inside a humidified incubator with 5% CO_2_ at 37 °C. When confluent, cells were serum-starved overnight (18 h) with DMEM (Invitrogen, Carlsbad, CA, USA) containing serum replacement media Insulin-Transferrin-Selenium (ITS, Invitrogen, Carlsbad, CA, USA) and 1% Pen/Strep (Lonza, Basel, Switzerland). Static mechanical stretch was applied for 24 h at a maximum distension of 23%. The control non-stretched (static) Flexcell plate was cultured inside the same incubator. Conditioned media (CM), both from non-stretched (NS) and stretched (S) plates, were collected, centrifuged for 10 min at 1000× *g*, 4 °C), and filtered through 0.22 mm Millex PEs syringe filter units (EMD Millipore Corp., Billerica, MA, USA) to remove cellular debris. Processed S-CM and NS-CM were kept at −20 °C until analysis.

### 2.4. Monocyte Harvesting and Differentiation

Peripheral blood (N = 6) was collected from third trimester pregnant women (37–40 weeks’ gestation). Monocytes were isolated using the RosetteSep Human Total Monocyte Enrichment cocktail (STEMCELL Technologies, Vancouver, BC, Canada) following the manufacturer’s protocol. In brief, the Monocyte RosetteSep cocktail with EDTA was added to whole blood (50 μL/mL of blood), incubated for 20 min at room temperature, and diluted with an equal volume of PBS containing 2% FBS. This mixture was carefully layered on top of Ficoll (Sigma-Aldrich, Oakville, ON, Canada) and centrifuged at 1200 rcf for 20 min. Isolated monocytes were washed twice with 15 mL of PBS, resuspended in RPMI cell culture media supplemented with 10% FBS (Wisent, Montreal, QC, Canada), counted, and plated at 5 × 10^6^ per 10 cm tissue culture plate (Eppendorf, Mississauga, ON, Canada). M1 polarization cells were differentiated for 6 days in media containing GM-CSF (100 ng/mL) and polarized for 2 days of LPS (100 ng/mL) and IFNγ (25 ng/mL); M2 polarization cells were differentiated for 6 days in M-CSF (100 ng/mL) and 2 days in IL-4 (25 ng/mL) and IL-3 (25 ng/mL) (Sigma-Aldrich, Oakville, ON, Canada) following established protocols [36]. Monocytes were also incubated in S-CM or NS-CM for 10 days. Media were changed every 2 days, and cells were visually monitored for morphological changes. Differentiated macrophages were harvested using a Macrophage Detachment Solution (PromoCell, Heidelberg, BW, Germany), washed with RPMI supplemented with 10% FBS (Wisent, Montreal, CQ, Canada), pelleted at 200 g for 5 min, counted, and used as needed.

### 2.5. Flow Cytometry

Fluorochrome-conjugated mouse anti-human antibodies CD14-PerCPCY5.5 (Cat# 562692), CDF68-APC (Cat# 560956), CD80-SB600 (Cat# 560925), and HLA-DR-PECF594 (Cat# 562304) were purchased from BD Biosciences (Mississauga, ON, Canada). Cells were detached using a Macrophage Detachment Solution (PromoCell, Heidelberg, BW, Germany), stained with a LIVE/DEAD™ Fixable Green Dead Cell Stain Kit (BD Biosciences, Mississauga, ON, Canada) in tandem with Human BD Fc Block™ (BD Biosciences, Mississauga, ON, Canada), washed (400 g, 6 min), stained with Abs in the dark for 1 h at RT, and resuspended with BD Stabilizing Fixative (BD Biosciences, Mississauga, ON, Canada). Stained samples were transferred into Fix/Lyse Solution (BD Biosciences, Mississauga, ON, Canada) and kept at 4 °C until flow cytometry analysis by 10-color Gallios Flow Cytometer (Beckman Coulter, Inc., Mississauga, ON, Canada). Monocytes and macrophages were identified by first gating based on the “forward and side scatter” area (which provides information on cell size and granularity), and then by using the monocyte-specific marker CD14 and the macrophage differentiation marker CD68 to distinguish monocytes from differentiated macrophages. To distinguish LIVE/DEAD cells, Alexa Fluor 488-Area (which binds to dead cells due to membrane permeability) was gated against the forward-side scatter area allowing the separation of live, intact cells from dead, permeable cells based on fluorescence and scatter properties. Doublet discrimination was achieved by gating forward scatter height versus area, as well as side scatter width versus height, to exclude cell clumps or doublets from analysis, as true single cells will show a proportional relationship between these parameters, while doublets will display disproportionate values. Data analysis was performed using KaluzaAnalysis Software v.1.2 (Beckman Coulter, Inc., Mississauga, ON, Canada). Compensation beads (BD Biosciences, Mississauga, ON, Canada) stained with each antibody were prepared and run with the samples in each experiment. The instrument was calibrated each time using Flow-Set Pro Fluorospheres (Beckman Coulter, Inc., Brea, CA, USA) before data acquisition to standardize fluorescent readings of individual experimental runs.

### 2.6. Cell Imaging

Following treatment of monocytes with SCM or NS-CM for 10 days, differentiated macrophages were incubated at RT for 1 h in HBSS−/− supplemented with 5µM of Cal-520/AM (Abcam, Waltham, MA, USA) for visualization in mobilized intracellular calcium, or with 5 µM of CM-H2DCFDA (Invitrogen, Carlsbad, CA, USA) for measurement of mROS superoxide. Cells were then rinsed three times in HBSS−/− and immediately imaged under different magnifications using Leica DM IL LED-inverted fluorescence microscope with a micropublisher 5.0 RTV Q imaging system or Leica Spinning Disc Confocal Microscope.

### 2.7. Protein Extraction and Immunoblotting

Human monocytes (N = 4) differentiated to macrophages for 10 days in NS-CM or S-CM with/without a BSCI were harvested and total protein was extracted using lysis buffer (0.08 M Tris/HCl -pH 6.8, 2% SDS, 10% Glycerol) supplemented with protease and a phosphatase inhibitor cocktail (Thermo Fisher Scientific Inc., Waltham, MA, USA). Protein concentration was determined by BCA (Thermo Fisher Scientific Inc., Waltham, MA, USA). An equal amount of protein was loaded to the gel with the first lane reserved for BLUelf Prestained Protein Ladder (GeneDireX, Taoyuan, Taiwan). Samples were separated by SDS-PAGE and transferred to a polyvinylidene difluoride (PVDF) membrane (Trans-blot Turbo Midi PVDF, BioRad, Mississauga, ON, Canada) using a Turbo Trans-Blot system (Bio-Rad, Mississauga, ON, Canada). Membranes were incubated on a rocker for 1 h in blocking solution (5% milk in TBST or 3% BSA (Wisent, Montreal, QC, Canada) in TBST), followed by incubation with primary antibodies diluted in the blocking solution at 4 °C overnight Phospho-NF-κB p65 Ser536 (Cat# 3033S) and NF-κB p65 (Cat# 8242S), Rabbit monoclonal 1:1000, (Cell Signaling, MA, USA); Annexin A1 (Cat# 71-3400) and Annexin A2 (Cat# PA5-27566), Rabbit polyclonal 1:1000, (Thermo Fisher Scientific Inc., Waltham, MA, USA). The membranes were washed thrice with TBS-T (for 10 min each) and subsequently probed with horseradish peroxidase-conjugated secondary antibody at room temperature for 1 h. Secondary anti-rabbit, anti-mouse, and anti-goat antibodies were purchased from Amersham (Thermo Fisher Scientific Inc., Waltham, MA, USA), 1:5000. Before visualization via chemiluminescent detection, membranes were incubated with SuperSignal West Femto Maximum Sensitivity Substrate (Thermo Fisher Scientific Inc., Waltham, MA, USA) at room temperature for 5 min. All immunoblots were imaged on the ChemiDoc Imaging System (Bio-Rad). To control for loading variations, membranes were stripped with RestoreTM PLUS Western blot stripping buffer (Thermo Fisher Scientific Inc., Waltham, MA, USA) and re-probed with anti-ERK2 antibody (Cat# ab227134) rabbit polyclonal dilution 1:1000 (Abcam, Waltham, MA, USA). Resulting images were scanned, analyzed, and normalized to the housekeeping protein ERK2. Quantification of protein expression was conducted using Image Lab Software (BioRad, Mississauga, ON, Canada).

### 2.8. Collagen Contractility Assay

The contractility assay was performed as described earlier [29]. Briefly, for the control monoculture experiment, confluent human myometrial cells (at least 3 individual primary cell lines at passages 3–5) were trypsinized and re-suspended in DMEM supplemented with 10% FBS (10% FBS-DMEM). For co-culture experiments, monocytes were treated with SCM or NS-CM for 10 days, harvested using enzyme-free Cell Dissociation Buffer (Thermo Fisher Scientific Inc., Waltham, MA, USA), and added to myocytes in a ratio of 1:10 (macrophages to myocytes). A rat tail collagen type I solution (3 mg/mL in 0.1 N HCl, Thermo Fisher Scientific Inc., Waltham, MA, USA) was adjusted to pH 7.2 with 0.1 N NaOH. Myocytes (or macrophage–myocyte suspension) were added to the neutralized collagen solution at a ratio of 1:1 for a total of 1 mL; the final concentration of collagen was 1.5 mg/mL. Collagen gel with cell suspensions were mixed in a 5 mL tube by inverting slowly until homogenized, and then carefully poured onto the well (100,000 cells/well) of the 12-well culture plate (Corning, Corning, NY, USA). Cell suspensions in collagen gel were incubated for 20 min at room temperature and then placed at 37 °C for 1 h to allow gelling. Subsequently, 1 mL of 10% FBS-DMEM was added over the collagen lattice. After 48 h, the culture medium was replaced with serum-free media (SFM). The lattices were gently detached from the sides and lifted off the bottom of the culture dish. Images of the floating gels were captured at “time 0” before detachment and then every day for up to 2 days and digitized using a ChemiDoc Imaging System (BioRad, Mississauga, ON, Canada); the area of the gels (mm^2^) was measured using Image J software. For each condition, collagen contraction was determined in 6 individual wells and averaged. Results were expressed as the percentage of contraction by calculating the difference in surface areas at time 0 versus 48 h.

### 2.9. Cell Viability Assay

At the end of gel contraction experiment, collagen lattices were placed in tubes containing 0.5 mL of a 0.1% collagenase solution (Sigma-Aldrich, Oakville, ON, Canada) and incubated for 10–20 min at 37 °C. Myometrial cells were pelleted by centrifugation at 200× *g* for 10 min and washed once with PBS−/−. Cells were stained with 0.4% Trypan Blue Solution (Thermo-Fisher Scientific Inc., Waltham, MA, USA) and viability was quantified using a hemocytometer.

### 2.10. Phagocytosis Assay

Human monocytes (N = 3) differentiated to macrophages for 10 days in NS-CM or S-CM with/without BSCI were seeded overnight at 5 × 10^5^ cells/well. The next day, the cells were pretreated with 20 μM Cytochalasin D (Abcam, Waltham, MA, USA) for 1 h at 37 °C before adding 5 μL of zymosan particles (Abcam, Waltham, MA, USA). Phagocytosis was conducted for 2 h, and the amount of engulfed zymosan was determined as recommended by manufacturer instructions. Fluorescent microscopy was performed using a Leica DM IL LED-Inverted fluorescence microscope with a micropublisher 5.0 RTV Q imaging system.

### 2.11. Mass Spectrometry Sample Preparation

Proteins were isolated from primary human stretched and non-stretched myocytes (N = 4) treated with/without the BSCI (400 nM), myocyte conditioned media (N = 4) concentrated using Amicon^®^ Ultra Centrifugal Filter, 100 kDa MWCO (Sigma-Aldrich, Oakville, ON, Canada), and macrophages treated by NS-CM or S-CM with/without the BSCI (N = 4). The digestion buffer contained 5% SDS and 50 mM triethylammonium bicarbonate, and 25 μg of protein material was reduced with 20 mM DTT for 10 min at 95 °C. Thereafter, the samples were alkylated with 40 mM iodoacetamide for 30 min in the dark. Samples were acidified by addition of phosphoric acid (1.2% final concentration). Then, 165 μL of S-Trap protein binding buffer (90% methanol, 100 mM TEAB) was added to 27.5 μL of the previously acidified lysate protein. The resulting mixture was passed through the micro column at 4000× *g*. The micro column was washed 3 times with the S-Trap protein binding buffer. Each sample was digested with 1 μg of trypsin (in 20 μL of 50 mM TEAB) for 1 h at 47 °C. Before elution, 40 μL of 50 mM TEAB pH 8 was added to the column. Peptides were eluted by centrifugation at 4000× *g*. Peptides were eluted 2 more times with 40 μL 0.2% formic acid and 40 μL of 50% acetonitrile + 0.2% formic acid. Eluted peptides were dried down and stored at −40 °C. Digested peptides were labeled with 80 μg of its respective TMTpro18 channel. Peptides were resuspended in 10 μL of 100 mM HEPES pH 8.0 and mixed with 80 μg TMTpro18 label resuspended in 4 μL of acetonitrile. This was incubated for 1 h at room temperature. The reaction was quenched with 4 μL of 5% hydroxylamine (diluted in 100 mM HEPES pH 8.0) for 15 min. Next, 82 μL of formic acid (5%) was added to each sample for a total volume of 100 μL. Then, 5 μL (1/20th) of each sample was combined into one TMT pool, which was then dried using a speed vac.

### 2.12. Mass Spectrometry Analysis

Analysis was performed in accordance with protocols from the https://nbcc.lunenfeld.ca/index.html, accessed on 4 March 2025. For data-dependent acquisition (DDA) LC-MS/MS, digested peptides were analyzed using a nano-HPLC (high-performance liquid chromatography) coupled to MS. A quarter of the combined TMT pool (pool = 1/20th of each sample) was used for DDA. Samples were separated on a Gen 3 Aurora Ultimate column (IonOpticks Pty Ltd., Collingwood, Victoria, Australia). The sample in 5% formic acid was directly loaded, with a combined control of flow rate 800 nL/minute and pressure limitation of 1200 bar, onto a 75 µm i.d.x 25 cm nano-spray emitter (packed with 1.7 µm C18 beads) heated at 40 °C with the TS Interface. Peptides were eluted from the column with an acetonitrile gradient generated by a Vanquish Neo UHPLC System (Thermo Fisher Scientific Inc., Waltham, MA, USA) and analyzed on an Orbitrap Fusion™Lumos™Tribrid™. The gradient was delivered at 400 nL/minute from 3.2% acetonitrile with 0.1% formic acid to 16.8% acetonitrile with 0.1% formic acid over 75 min, 16.8% to 24.8% acetonitrile with 0.1% formic acid over 28 min, and 24.8% to 35.2% acetonitrile with 0.1% formic acid over 17 min. This was followed by a column wash of 76% acetonitrile with 0.1% formic acid over 13 min. The LC method ends with an equilibration of 4 column volumes at the same combined control as loading. The total DDA protocol was 135 min. The MS1 scan had an accumulation time of 50 ms within a scan range of 400–1600 *m*/*z*, using an orbitrap resolution of 120,000, 30% RF lens, AGC target of 200%, and 1800 volts. This was followed by MS/MS scans with a total cycle time of 2 s. An accumulation time of 35 ms and 35% HCD collision energy was used for each MS/MS scan, ranging from 120 to 1800 *m*/*z*. Each candidate ion was required to have a charge state from 2 to 7 and an AGC target of 300%, isolated using an orbitrap resolution of 50,000. Previously analyzed candidate ions were dynamically excluded for 9 s.

### 2.13. Mass Spectrometry Data Analysis

The RAW files were searched with FragPipe v20.0, using MSFragger v3.8 and Philosopher v5.0.0. The TMT16 workflow with the Homo Sapiens Uniprot ID UP000005640 database (with decoys and contaminants appended) was utilized. Oxidation of methionine (15.9949), terminal acetylation (42.0106), and N-terminus modified by the TMT reactive group (304.20715) were set as variable modifications. Lysine modified by the TMT reactive group (304.20715) was set as a fixed modification. Precursor mass tolerance was set to 40 ppm. PSM Validation cmd line: --only-psms --no-terminate --post-processing-tdc; ProteinProphet cmd line: --maxppmdiff 2,000,000 --minprob 0.5; FDR filter and report cmd line: --sequential --picked --prot 0.01. All other parameters were set to their default configuration.

### 2.14. Bioinformatics Analysis

Gene ontology (GO) enrichment and Kyoto Encyclopedia of Genes and Genomes (KEGG) pathway analyses of the significantly different proteins between groups were performed using ClueGO plug-in and Cluepedia of Cytoscape software 3.10.3. The results were filtered with the thresholds of count >2 and *p*-value < 0.05.

### 2.15. Statistical Analysis

The normality of datasets was determined by the Shapiro–Wilk test. The Grubbs’s outliers test was implemented with the assumption of a normally distributed population. Mann–Whitney test (two-tailed), Pearson correlation, Chi-square analysis, Kaplan–Meier analysis (Log-rank), paired two-tailed Student’s *t*-test (for protein expression difference in Proteomics experiments), or unpaired two-tailed Student’s *t*-test (two groups) or two-way ANOVA (more than two groups) were used as appropriate. The data are presented as the means ± SEMs. The significance level was set at *p* < 0.05 (*), *p* < 0.01 (**), and *p* < 0.001 (***).

## 3. Results

### 3.1. Mass Spectrometry Analysis of Proteins Secreted by Stretched Myometrial Cells

**Figure 1:**
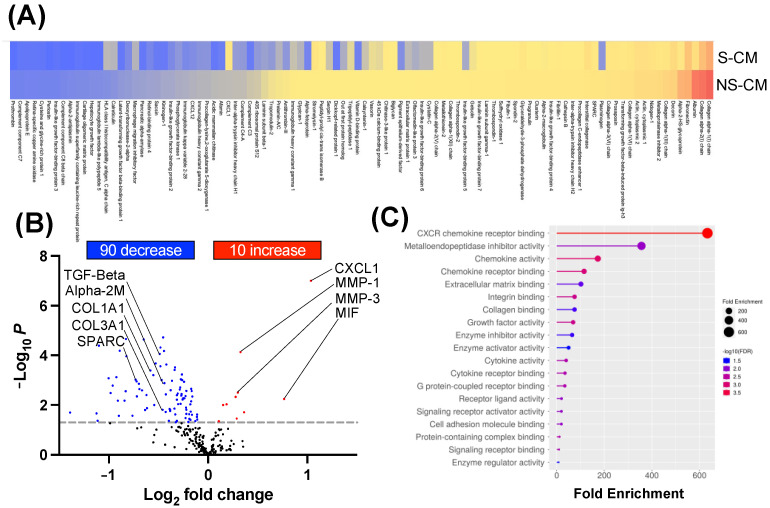
Proteomic analysis of proteins secreted by stretched primary human myocytes. Myometrial biopsies from pregnant term non-laboring women (N = 4/group) were used to isolate primary uterine myocytes subjected to mechanical stretch (23% elongation, 24 h) using a Flexcell instrument. Conditioned media (CM) from stretched (S-CM) and non-stretched (NS-CM) myocytes were collected, concentrated, and analyzed by TMT-MS to identify differentially secreted proteins. (**A**) Heatmap displaying secreted proteins significantly modulated by stretch (S-CM vs. NS-CM), with red indicating high abundance and blue indicating low abundance. (**B**) Volcano plot highlighting differentially secreted proteins, showing significantly increased and decreased proteins (upregulated: red; downregulated: blue) in response to stretch. (**C**) Gene ontology (GO) enrichment analysis of the top 20 biological processes affected by mechanical stretch, with circle size representing the number of enriched genes and color indicating significance (larger circles indicate higher gene enrichment; higher significance in red). Statistical significance was determined by two-sided unpaired Student’s *t*-tests.

Conditioned media (CM) were collected from non-stretched (NS-CM) and stretched (S-CM) primary human myometrial cells after undergoing 24 h of 23% static stretch (N = 4/group). CM were concentrated and analyzed by tandem mass spectrometry (TMT-MS) to determine their composition. The heatmap showed 100 secreted proteins differentially regulated between NS-CM and S-CM (*p* < 0.05–0.001, Figure 1A), with 90 protein intensities being decreased and 10 proteins increased in S-CM relative to NS-CM (Figure 1B). Gene ontology enrichment analysis (biological process) showed that biological processes significantly (*p* < 0.05–0.001) affected by static stretch in human myocytes included CXCR chemokine activity, cytokine activity, growth factors, extracellular matrix (ECM) binding, and collagen binding (Figure 1C). Notably, the most decreased proteins were related to the ECM remodeling process, while increased proteins belonged to inflammatory pathways.

### 3.2. Monocytes Treated with Myometrial S-CM Are Differentiated and Polarized into M1 Macrophages

**Figure 2:**
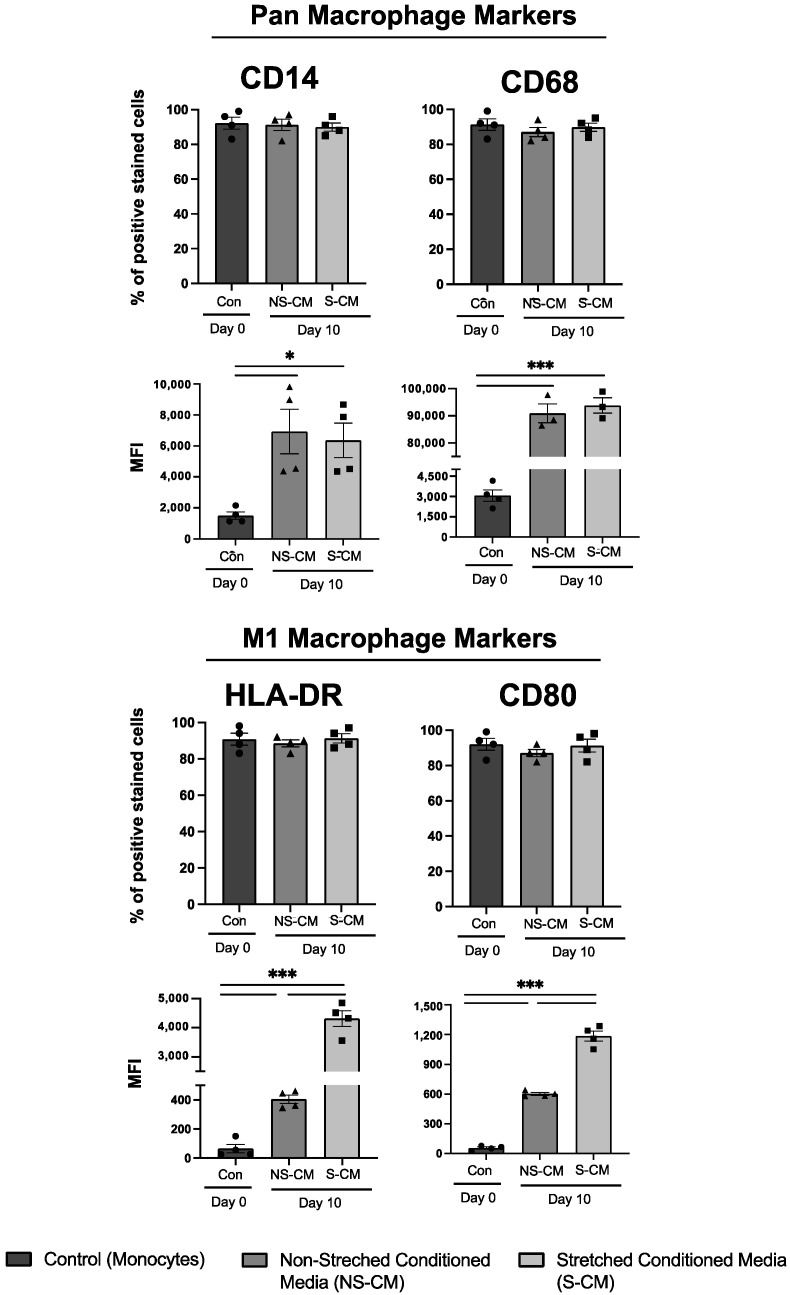
Differentiation of human peripheral monocytes into macrophages and their polarization in response to conditioned media (CM) from stretched (S-CM) or non-stretched (NS-CM) human uterine myocytes. Monocytes from pregnant term non-laboring women (N = 3–4/group) were cultured for 10 days in CM. Flow cytometry analysis assessed CD14 (monocyte marker), CD68 (macrophage marker), and M1 polarization markers (HLA-DR, CD80). Bar graphs display the percentage of positively stained cells and mean fluorescence intensity (MFI). Statistical significance was determined by one-way ANOVA with Dunnett’s test (* *p* < 0.05, *** *p* < 0.001).

Following treatment with NS-CM and S-CM for 10 days, primary human monocytes (N = 3–4/group) isolated from term pregnant women were differentiated into macrophages as manifested by a significant increase in the mean fluorescence intensity (MFI) of the CD14 and CD68 proteins (*p* < 0.05 and *p* < 0.001, respectfully) (Figure 2). NS-CM and S-CM both significantly (*p* < 0.001) induced the expression (MFI) of M1 macrophage markers (HLA-DR and CD80) compared to monocyte controls, with S-CM being 2–10 times the more potent inducer of pro-inflammatory M1 phenotype compared to NS-CM. These flow cytometry results indicate that the incubation for 10 days was sufficient to achieve both the differentiation of monocytes to macrophages and their polarization to an M1 phenotype (gating strategy is shown in Supplemental Figure S1).

**Figure 3:**
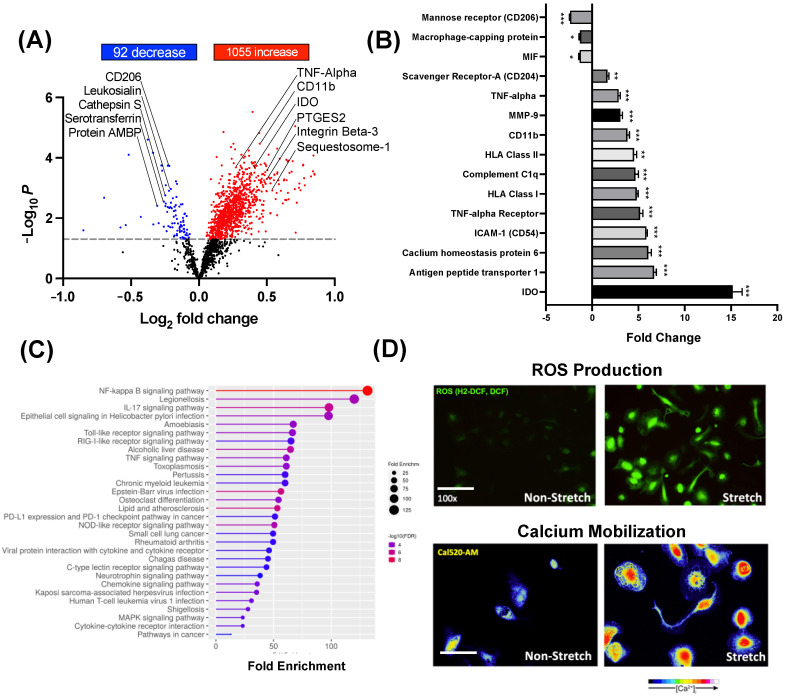
Proteomic profiling of macrophages activated by myocyte-secreted factors. Human monocytes (N = 4/group) were treated for 10 days with conditioned media (CM) from stretched (S-CM) or non-stretched (NS-CM) human myocytes, then analyzed by TMT-MS to assess macrophage polarization. (**A**) Volcano plot showing differentially expressed proteins in S-CM vs. NS-CM-treated macrophages (upregulated: red; downregulated: blue). (**B**) Bar graph of key protein markers significantly altered by S-CM (mean ± SD; Student’s *t*-test; * *p* < 0.05, ** *p* < 0.01, *** *p* < 0.001). (**C**) GO enrichment analysis of the top 20 biological processes affected in S-CM-treated macrophages with circle size representing the number of enriched genes and color indicating significance (larger circles indicate higher gene enrichment; higher significance in red). (**D**) Fluorescence imaging of S-CM- and NS-CM-treated macrophages stained for ROS production (H2-DCF, green) and Ca^2^⁺ mobilization (Cal-520 AM, yellow), captured by confocal microscopy.

Next, monocytes (N = 4/group) were treated for 10 days with S-CM or NS-CM, and differentiated immune cells were analyzed by TMT-MS. Results indicate that 92 proteins were significantly decreased and 1.055 significantly increased in macrophages treated with S-CM compared to NS-CM (Figure 3A, *p* < 0.05–0.001). In particular, we observed a significant (*p* < 0.001) increase in pro-inflammatory markers: Indoleamine 2,3-dioxygenase (15-fold), TNF-α (2.9-fold), TNF-α receptor (5.2-fold), Integrin β-3 (14-fold), ICAM-1 (5.9-fold), HLA Class I (5.2-fold), Complement C1q (4.7-fold), HLA Class II (4.5-fold), Matrix metalloproteinase-9 (3.1-fold), Antigen peptide transporter 1 (6.7-fold), Toll-like receptor-4 (2.7-fold), and Calcium homeostasis modulator protein 6 (6.1-fold) in macrophages treated with S-CM compared to NS-CM (Figure 3B). Gene ontology enrichment analysis (biological process) identified upregulation of NF-κB signaling, TNF signaling, TLR signaling pathways, and cytokine/chemokine pathways in macrophages treated with S-CM (Figure 3C). Moreover, to confirm the ability of S-CM to promote pro-inflammatory M1 polarization of human macrophages, we stained the cells with a marker of reactive oxygen species (H2-DCF) and with the mobilized calcium marker (Cal520-AM). The intensity of both markers was higher in macrophages treated with S-CM as compared to NS-CM (Figure 3D).

### 3.3. BSCI-Treated Human Myocytes Secrete Tissue-Remodeling Proteins

**Figure 4:**
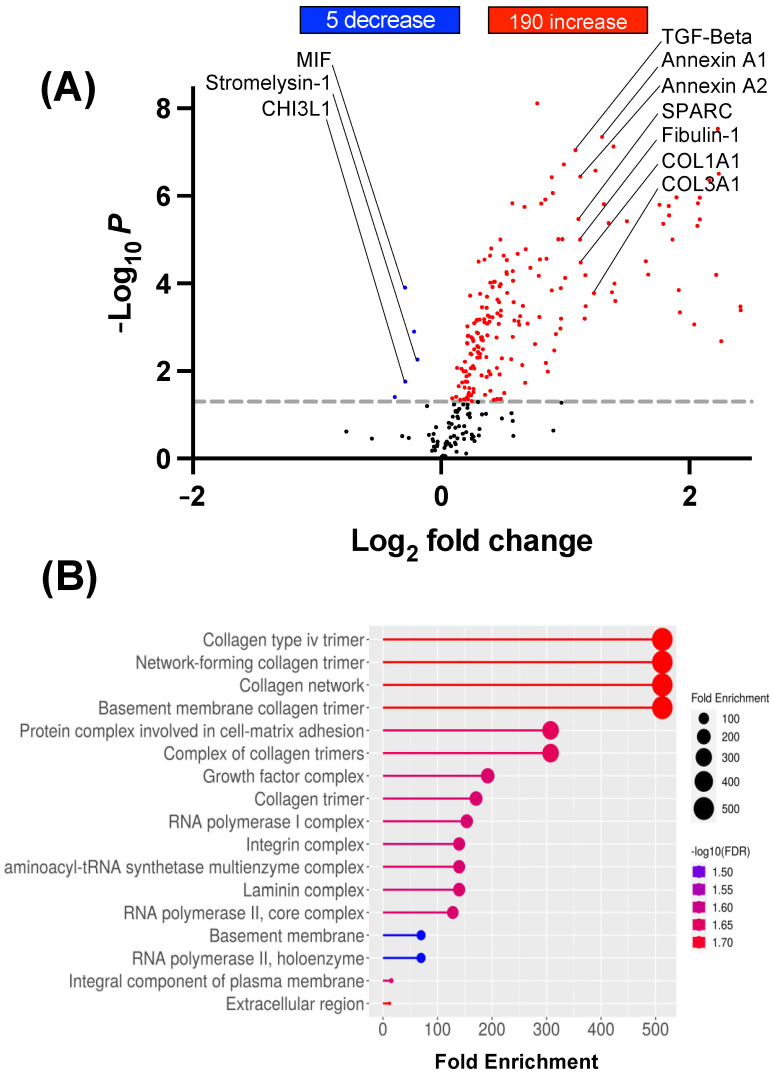

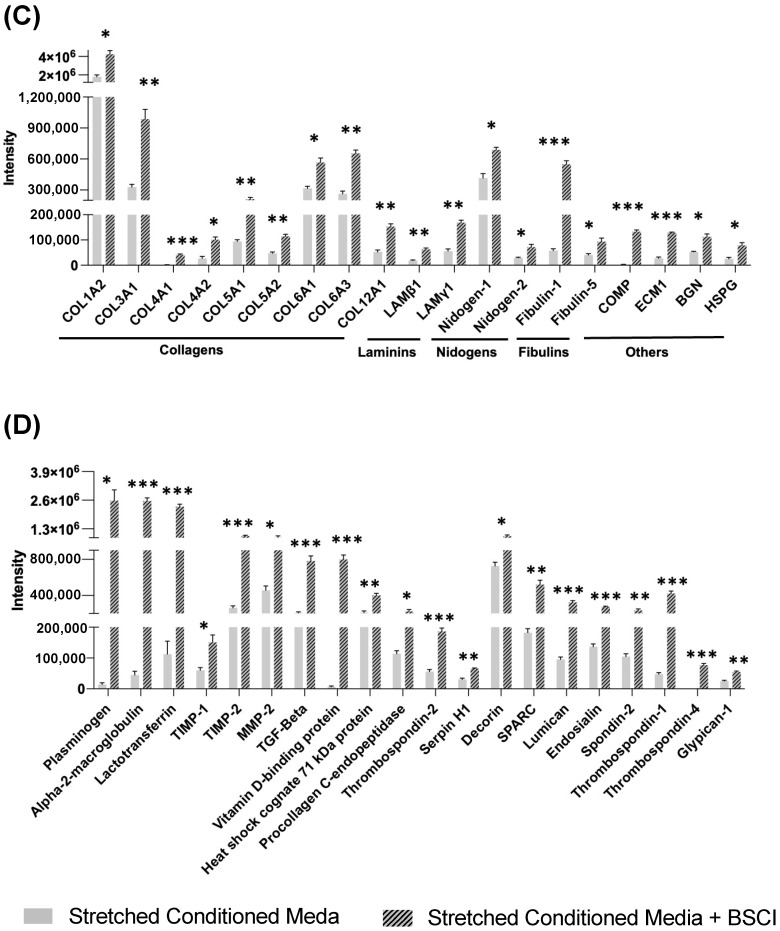
Proteomic analysis of secreted proteins from BSCI-treated stretched myocytes. Primary human myocytes (N = 4/group) from term pregnant, non-laboring women were pretreated with BSCI (400 nM, 1 h) before undergoing mechanical stretch (23% elongation, 24 h). Conditioned media (CM) from stretched myocytes (S-CM) and BSCI-treated stretched myocytes (S-CM + BSCI) were collected and analyzed by TMT-MS to assess changes in secreted protein composition. (**A**) Volcano plot showing differentially secreted proteins in S-CM + BSCI vs. S-CM (upregulated: red, downregulated: blue). (**B**) GO enrichment analysis of the top 20 biological processes modified by BSCI treatment with circle size representing the number of enriched genes and color indicating significance (larger circles indicate higher gene enrichment; higher significance in red). (**C**) Extracellular matrix (ECM)-associated proteins upregulated in BSCI-treated myocytes, suggesting enhanced tissue remodeling. (**D**) Key tissue remodeling proteins increased following BSCI treatment, indicating a shift toward an anti-inflammatory, reparative secretome. Statistical significance: two-sided unpaired Student’s *t*-tests (* *p* < 0.05, ** *p* < 0.01, *** *p* < 0.001).

To determine the effect of BSCIs on the myocyte secretome, primary human myometrial cell lines (N = 4/group) were pretreated for 1 h with the drug before stretch. The BSCI (400 nM) remained in the cell culture media during the 24 h static stretch (S-CM + BSCI). Conditioned media were collected, concentrated, and analyzed by TMT-MS. The heatmap shows 195 secreted proteins were differentially regulated between S-CM and S-CM + BSCI, with 190 protein intensities increased and five proteins decreased in S-CM + BSCI relative to S-CM (*p* < 0.05–0.001, Figure 4A). Furthermore, gene ontology enrichment analysis (biological process) showed that pathways with the most significant (*p* < 0.001) change between S-CM + BSCI and S-CM were related to ECM remodeling, including ECM-matrix binding and collagen synthesis (Figure 4B). Notably, ECM components (Collagen I, Collagen III, Collagen VI, *p* < 0.001, 2.3- to 3.0-fold increase), ECM-associated proteins (Decorin, Cartilage oligomeric matrix protein, Lumican, 2- to 68-fold increase, *p* < 0.001), and tissue repair and wound healing proteins promoting M2 macrophage phenotype (Plasminogen, TGF-β, α2-Macroglobulin, 16- to 180-fold increase, *p* < 0.001) were significantly upregulated (Figure 4C,D).

### 3.4. Myocyte BSCI Treatment Drives Homeostatic M2 Macrophage Polarization

To determine if the BSCI could alter the monocyte-to-macrophage transition, we treated primary human monocytes (N = 3–4/group) with S-CM + BSCI (400 nM) versus S-CM alone. Flow cytometry results indicate a significant (*p* < 0.001) decrease in M1 macrophage markers CD80 and HLA-DR, but not CD68, suggesting that the BSCI indirectly alters macrophage polarization without changing the differentiation state. Moreover, direct treatment of monocytes with the BSCI did not alter their differentiation (Supplemental Figure S2); it was rather achieved indirectly by treatment with the myocyte secretome.

**Figure 5:**
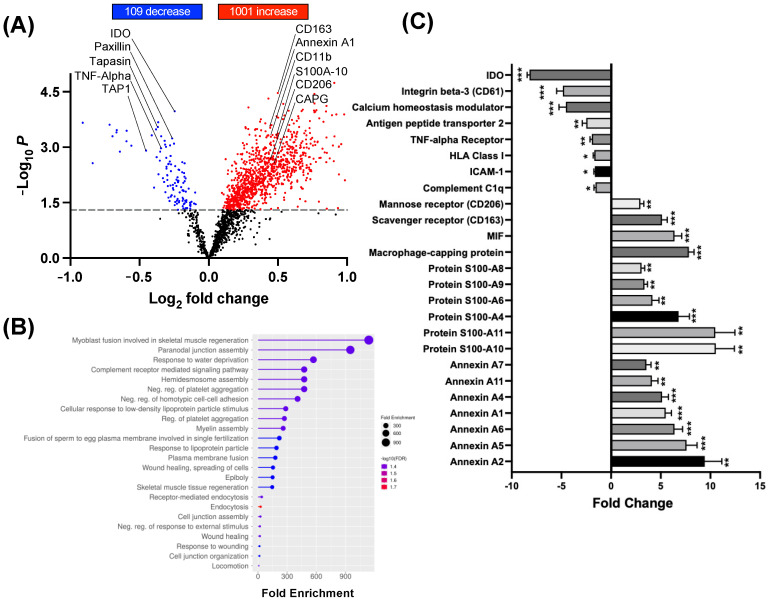
Proteomic analysis of macrophages exposed to secretome from BSCI-treated myocytes. Primary monocytes (N = 4/group) were cultured for 10 days in conditioned media (CM) from mechanically stretched uterine myocytes (S-CM) pretreated with or without a BSCI (400 nM, 1 h). Total protein content of differentiated macrophages was analyzed using TMT-MS. (**A**) Volcano plot displays differentially expressed proteins in macrophages treated with S-CM vs. S-CM + BSCI (upregulated: red; downregulated: blue). (**B**) Gene ontology (GO) enrichment analysis of the top 20 biological processes altered by the BSCI, with circle size representing the number of enriched genes and color indicating significance (larger circles indicate higher gene enrichment; higher significance in red). (**C**) Bar graph shows fold changes in selected protein markers affected by BSCI treatment. Statistical significance was determined by two-sided unpaired Student’s *t*-tests (* *p* < 0.05, ** *p* < 0.01, *** *p* < 0.001).

To examine the proteomic profile of macrophage treated with S-CM + BSCI versus S-CM without the BSCI, cell pellets were collected after 10 days, and analyzed by TMT-MS (N = 4/group). Results identified 109 proteins significantly (*p* < 0.05–0.001) decreased and 1.001 significantly (*p* < 0.05–0.001) increased in macrophages treated with S-CM + BSCI compared to S-CM (Figure 5A). Gene ontology enrichment analysis (biological process) showed that anti-inflammatory pathways were significantly (*p* < 0.05–0.001) induced in macrophages by BSCI treatment. These included myoblast fusion proteins involved in skeletal muscle regeneration, wound healing, muscle tissue regeneration, endocytosis, and others (Figure 5B). Importantly, we observed a significant (*p* < 0.001) increase in anti-inflammatory markers CD206 (2.9-fold), CD163 (5.1-fold), MIF (6.4-fold), CD31 (2.6-fold), macrophage-capping protein (7.8-fold), and Annexin proteins—A1 (5.5-fold), A2 (9.4-fold), and A4 (5.1-fold)—in S-CM + BSCI-treated macrophages compared to S-CM-treated cells (Figure 5C).

**Figure 6:**
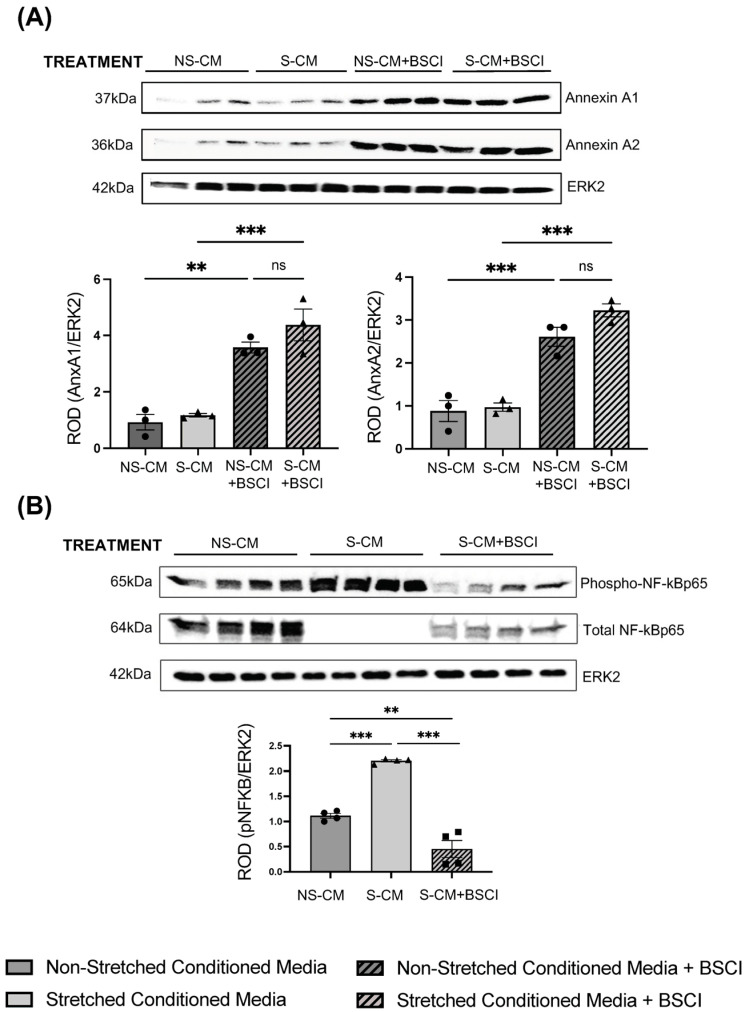
Validation of protein expressed by human macrophages exposed to myocyte secretome and treated with BSCI. Primary human monocytes (N = 3–4/group) were cultured for 10 days in conditioned media (CM) from non-stretched (NS-CM), mechanically stretched (S-CM), and BSCI-treated myocytes (400 nM, 1 h) (NS-CM + BSCI, S-CM + BSCI). Total protein was extracted from differentiated macrophages and analyzed by Western blot. (**A**) Representative Western blots and densitometric analysis of Annexin A1 and Annexin A2 expression, normalized to ERK2, showing differential expression in response to mechanical stretch and BSCI treatment. (**B**) Expression of phospho-NF-κBp65 and total NF-κBp65 in macrophages treated with NS-CM, S-CM, or S-CM + BSCI. Densitometric analysis quantifies phospho-NF-κBp65 levels relative to ERK2, indicating changes in NF-κB signaling in response to myocyte-derived factors and the BSCI. Data are presented as mean ± SD, with statistical significance determined by one-way ANOVA followed by Dunnett’s multiple comparisons test (** *p* < 0.01, *** *p* < 0.001, “ns”—non significant).

We validated our TMT-MS results by immunoblotting using total proteins extracted from macrophages (N = 3–4/group) pretreated with CM with/out the BSCI (400 nM) and confirmed that Annexin A1 and A2 were significantly (*p* < 0.01–0.001) increased in immune cells treated with NS-CM + BSCI or S-CM + BSCI, regardless of myocyte stretch status (Figure 6A). To explore the intracellular signaling pathway regulating Annexin proteins, which play a significant role in the resolution of inflammation, we examined the phosphorylation of the transcription factor NF-κB. Mechanical stretch indirectly activated NF-κB in macrophages through myocyte secretome (NS-CM versus S-CM, *p* < 0.001), which was counteracted by myocyte treatment with the BSCI (400 nM) (Figure 6B).

**Figure 7:**
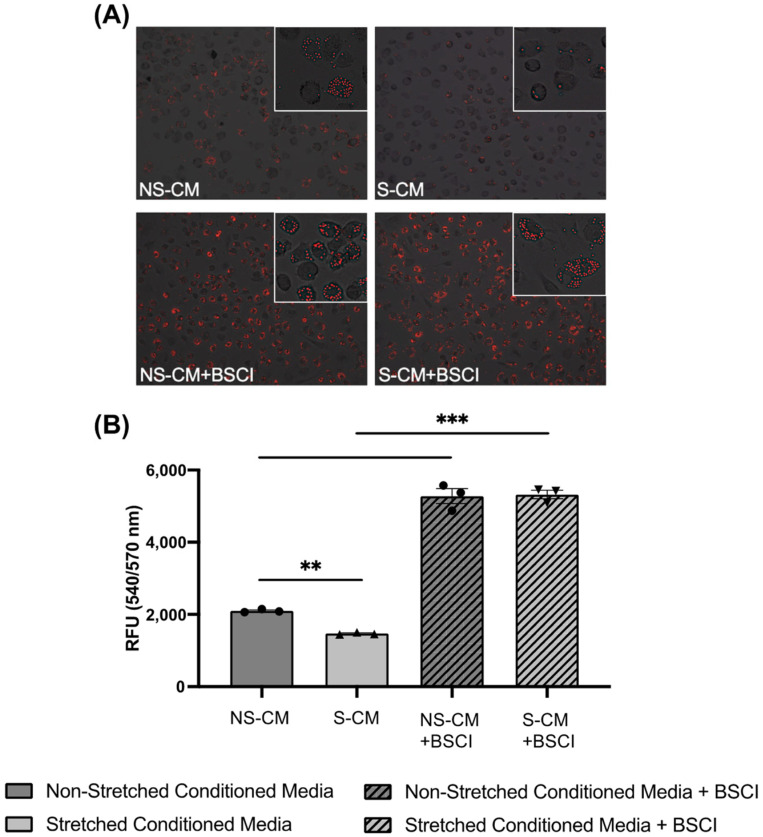
Enhanced phagocytosis in macrophages exposed to BSCI-treated myocyte secretome. Monocytes isolated from pregnant term non-laboring women (N = 3/group) were differentiated into macrophages over 10 days in conditioned media (CM) from four groups: (1) non-stretched myocytes (NS-CM), (2) mechanically stretched myocytes (S-CM), (3) non-stretched myocytes pretreated with BSCI (NS-CM + BSCI), and (4) stretched myocytes pretreated with BSCI (400 nM, 1 h) (S-CM + BSCI). After differentiation, macrophages (5 × 10^4^) were incubated with zymosan particles at 37 °C for 1 h, and phagocytosis was assessed by real-time confocal fluorescence microscopy. (**A**) Representative immunofluorescent images of zymosan uptake by macrophages. (**B**) Quantification of relative fluorescence units (RFUs) from duplicate plate readings. Data represent the mean ± SD of three independent macrophage lines (N = 3/group). Statistical significance was determined by one-way ANOVA followed by Dunnett’s multiple comparisons test (** *p* < 0.01, *** *p* < 0.001). Original magnification: ×20; inset magnification.

We hypothesized that the BSCI-treated myocyte secretome (S-CM + BSCI) could change the polarization of macrophages from M1 to an M2-like phenotype, which is characterized by increased phagocytotic activity [37]. Human monocytes were differentiated and polarized for 10 days to pro-inflammatory M1 (GM-CSF + LPS, IFNγ) or anti-inflammatory M2 (M-CSF + IL-13, IL-4) macrophages. The control experiments confirmed a significantly higher level of phagocytosis by M2 compared to M1 cells (Supplemental Figure S3). Next, we performed a phagocytosis assay using macrophages treated with control NS-CM or S-CM. Results complimented our TMT-MS data, suggesting that S-CM treated macrophages were more M1-like as demonstrated by significantly (*p* < 0.01) decreased phagocytosis as compared to NS-CM macrophages (Figure 7A,B). Furthermore, macrophages treated with CM with/without the BSCI (400 nM) were found to have ~three times more phagocytotic activity with a significant (*p* < 0.01–0.001) increase following drug treatment.

### 3.5. The BSCI Prevents the Contraction of Collagen Gel Myocyte–Macrophage Co-Cultures

**Figure 8:**
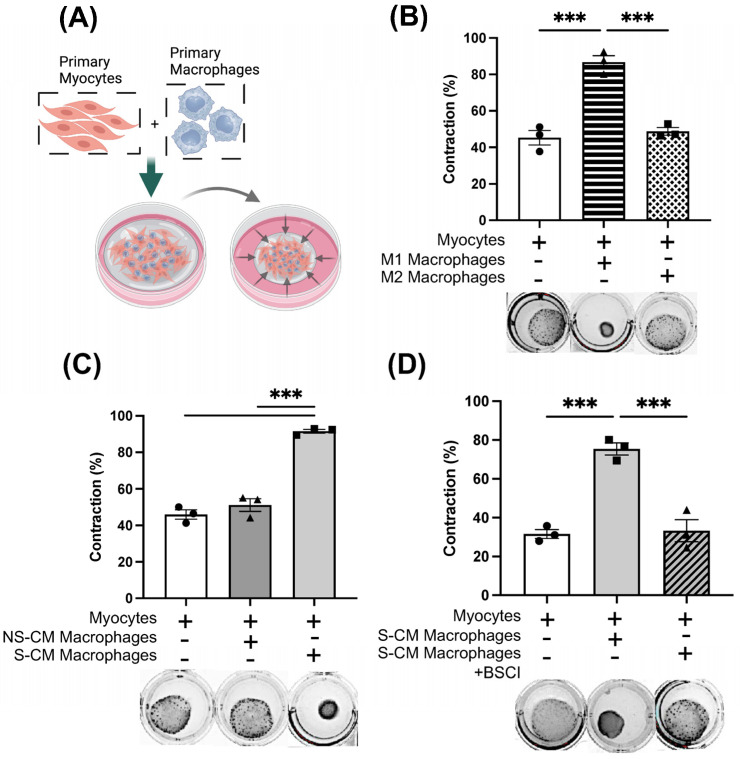
The BSCI inhibits collagen lattice contraction in myocyte–macrophage co-cultures. (**A**) Schematic of the co-culture treatment protocol. Monocytes and uterine smooth muscle cells were isolated from pregnant term non-laboring women (N = 3/group). Monocytes were differentiated for 10 days before co-culturing with myocytes in a collagen gel lattice, and gel contraction was measured after 48 h. (**B**) Collagen gel contraction induced by myocyte co-culture with M1 (GM-CSF, LPS, IFNγ) or M2 (M-CSF, IL-13, IL-4) macrophages, compared to myocyte monoculture (negative control). (**C**) Collagen contraction in co-cultures of myocytes with macrophages treated with conditioned media (CM) from non-stretched (NS-CM) and mechanically stretched (S-CM) myocytes, compared to myocyte monoculture. (**D**) Effect of the BSCI on collagen contraction. Macrophages treated with CM from stretched myocytes (S-CM) with or without BSCI pretreatment (400 nM, 1 h) were co-cultured with myocytes. Differences in gel surface area were recorded at 48 h. Six wells per condition were used for each primary co-culture (N = 6, technical replicates), with three myometrial cell lines analyzed (N = 3/group). Data are presented as mean ± SD. Statistical significance was determined by one-way ANOVA with Dunnett’s multiple comparisons test and two-sided unpaired Student’s *t*-tests (*** *p* < 0.001).

Next, we explored the ability of the BSCI to influence the contractility of smooth muscle cells. Human monocytes (N = 3/group) were differentiated and polarized to pro-inflammatory M1 or anti-inflammatory M2 macrophages as described in methods, co-cultured with primary human myocytes in collagen gel lattices and allowed to contract for 48 h (Figure 8A). The myocyte contractile ability in co-culture was quantified as the percentage of gel area reduction between 0 and 48 h. Myo-M1 co-cultures induced significantly more gel contraction than Myo-M2 co-cultures and control myocyte cultures (Figure 8B, 87% Myo-M1 versus 45% Myo-M2, *p* < 0.001). Next, macrophages (N = 3/group) treated with S-CM or NS-CM for 10 days were co-cultured with uterine myocytes for 48 h in gel lattices. Results indicated that S-CM-treated macrophages induced significant gel contraction as compared to NS-CM-treated cells (Figure 8C, 92% Myo-S-CM-Mac versus 51% Myo-NS-CM-Mac, *p* < 0.001). Next, we examined whether BSCI treatment would indirectly (via modified secretome of drug-treated myocytes) re-polarize macrophages to anti-inflammatory M2 phenotype, thus influencing myocyte contractility. We treated monocytes (N = 3/group) for 10 days in S-CM + BSCI and co-cultured differentiated macrophages with uterine myocytes for 48 h in gel lattices. Our results indicated that when macrophages were treated with S-CM + BSCI, gel contraction of Myo-Mac co-cultures was significantly less compared to Myo-S-CM-Mac (Figure 8D, 78% Myo-S-CM-Mac versus 42% Myo-S-CM-Mac + BSCI, *p* < 0.001). Based on these results, we speculate that S-CM can polarize macrophages to a phenotype similar to that of pro-inflammatory M1, which promotes uterine myocyte contractility. At the same time, these data suggest that the BSCI could modulate the myocyte secretome, thus changing the phenotype of macrophages to a state similar to M2 and consequently preventing myocyte contractility. To exclude the effect of the BSCI on cell viability, at the end of the experiments, gel lattices were treated with collagenase to recover and count cells. Results indicated similar cell viability between treatment groups, with no significant deviations (Supplemental Figure S4).

## 4. Discussion

The findings presented in this study offer novel insights into the complex interplay between uterine myocytes and macrophages and their roles in regulating inflammation associated with myometrial contractility. Our results demonstrate that stretched human uterine myocytes secrete a unique set of proteins that drive monocyte differentiation and polarization toward a pro-inflammatory M1-like phenotype. This suggests that the mechanical stretch of the uterine walls by the growing fetus, a defining feature of late gestation, may play a pivotal role in directly shaping the local macrophage environment in the uterus in preparation for labor onset. Moreover, we have discovered that a novel therapeutic BSCI (FX125L) can modulate myocyte protein secretion, promoting the release of anti-inflammatory tissue-remodeling proteins indirectly influencing macrophage polarization.

Our findings align with the growing body of literature that highlights the importance of macrophages in pregnancy maintenance and the potential of targeting these immune cells to prevent PTB [16,18,23,24,26,38]. Previous studies have demonstrated the presence of both M1 and M2 macrophages in the uterine tissues during pregnancy, with a shift toward M1 polarization and a decrease in M2 macrophages associated with PTL [23,24,25,39]. However, the specific mechanisms that govern this shift in the local uterine macrophage polarization have remained unexplained. Our previous in vitro research using multiplex antibody-based technology showed that static mechanical stretch of immortalized human myocytes induced the secretion of multiple pro-inflammatory cytokines, chemokines, and growth factors, including interleukin-6 (IL-6), IL-12p70, Macrophage migration inhibitory factor (MIF), Chemokine (C-X-C motif) ligand 1 (CXCL1), Chemokine (C-X-C motif) ligand 8 (CXCL8), Granulocyte colony stimulating factor (G-CSF), Vascular endothelial growth factor (VEGF), basic Fibroblast growth factor 2 (bFGF), and Platelet-derived growth factor two B subunits (PDGF-BB) [10]. All of these factors influence the activation and recruitment of peripheral leukocytes into the myometrium [7,9,10,35]. In the current study, we used an unbiased, high-throughput methodology of protein mass spectrometry to examine the secretion of primary human myometrial cells following 24 h of static mechanical stretch. This approach allowed us to analyze a much higher range of already-known proteins and various new molecules secreted by stretched human myocytes. Consistent with our previous data [10], we observed the chemokines CXCL1 and MIF were most highly increased in the conditioned media of stretched myocytes. CXCL1 is well known for its strong chemoattractant activity on neutrophils, and its expression in human myometrium has been noted by many research groups [10,40,41]. CXCL1 further amplifies the immune response by promoting the production of pro-inflammatory cytokines and chemokines, such as TNF-α, IL-1β, IL-6, and Chemokine (C-C motif) ligand 2 (CCL2), creating feedback loops that sustain and intensify inflammation [42,43,44,45]. MIF is a pro-inflammatory cytokine that promotes immune cell activation, enhances the production of other pro-inflammatory cytokines, and can modulate T-cell responses [46], as well as T-cell–macrophage interactions [47,48,49]. Gene ontology analysis further revealed CXCR chemokine receptor binding as the highest enriched pathway based on all significantly differentiated secreted proteins, emphasizing the impact of pro-inflammatory cytokine and chemokine signaling within the secretome. Together, these data further emphasize the pro-inflammatory nature of the secretome of stretched uterine myocytes.

Interestingly, we found a dramatic decrease of 90 secreted proteins by stretched myocytes, the majority associated with extracellular matrix (ECM) tissue remodeling. We have reported before that uterine smooth muscle cells are highly plastic and undergo a series of phenotypic changes throughout pregnancy [11,50,51,52,53,54,55]. We have shown that these changes include an early proliferative phase, an intermediate synthetic phase, a contractile phase, and a final labor phase [11,52]. The synthetic phase of myometrial differentiation in vivo is characterized by myocyte cellular growth, increased ECM elaboration, and changes in cell–matrix interactions [11,52]. Our results indicate that it is also associated with increased expression of insulin-like growth factor binding protein 6 (IGFBP6) [53]. Contractile smooth muscle cells, when cultured in vitro following isolation, de-differentiate and acquire a synthetic phenotype [56,57,58,59]. Our current in vitro data suggest that static mechanical stretch influenced the phenotype of cultured primary human myocytes toward that of a contractile state. This was evidenced by a significant decrease in ECM proteins (i.e., Periostin, Fibrillin-1, Decorin, Biglycan, Cartilage oligomeric matrix protein (COMP), Extracellular matrix protein 1 (ECM1)) as well as a decrease in major growth factors (i.e., Hepatocyte growth factor (HGF), TGF-β, and Pigment epithelium-derived factor (PEDF)), all of which are related to the maintenance and synthesis of the ECM [60,61,62]. Notably, the major myometrial fibrillar collagens, Collagen 1 and Collagen 3 [54], and proteins involved in collagen synthesis (i.e., Serpin H1, Procollagen c-endopeptidase enhancer 1, Secreted protein acidic and rich in cysteine/SPARC) were decreased in stretched myocytes. Furthermore, MMP-1 and MMP-3, the major tissue enzymes involved in the breakdown of ECM, were highly upregulated, while IGFBP6 was significantly decreased. These data suggest a possible transition of myometrial cells from synthetic to contractile phenotype due to mechanical stretching.

Next, we sought to investigate the effects of the myometrial secretome on monocyte differentiation and macrophage polarization. Using flow cytometry, we found that after 10 days in culture, monocytes treated with either S-CM or NS-CM were differentiated into macrophages, as seen from an increase in macrophage-specific markers CD68 and CD14. Furthermore, after treatment with NS-CM or S-CM, macrophages were polarized to M1 as manifested in an increase in M1 markers, MHC class II markers (HLA-DR), and costimulatory signal receptor CD86. Importantly, S-CM-treated macrophages had a significantly higher expression of both M1 markers compared to the NS-CM-treated macrophages. This prompted a future study into the immunophenotype of CM-treated macrophages beyond the classical M1 protein markers.

Using protein mass spectrometry, we discovered the unique pro-inflammatory profile of S-CM-treated compared to NS-CM-treated macrophages. This was manifested by an increase in TNF-α, and receptor TNF-αR, MMP9, CD11b, HLA-DR, Complement C1q, ICAM-1, Antigen peptide transporter 1, IDO, and a decrease in the M2 macrophage receptor CD206 and chemokine MIF. Furthermore, gene ontology analysis demonstrated that the pro-inflammatory NF-κB signaling pathway was highly enriched in the S-CM-treated macrophages compared to NS-CM-treated macrophages. In addition, live cell imaging showed a dramatic increase in ROS production and Ca^2+^ mobilization in S-CM-treated macrophages, both of which are elevated in M1 macrophages [54,63,64]. These data further validate our hypothesis that the mechanical stretch of uterine myocytes induces the secretion of components that drive a pro-inflammatory M1 macrophage phenotype.

Since stretched myocytes were the source of pro-inflammatory signals driving M1 macrophage polarization, we investigated whether the BSCI could target the myocytes themselves. Using protein mass spectrometry, we examined the secretome of BSCI-treated stretched myocytes. We found a remarkable switch in the secretome of BSCI-treated stretched myocytes from a pro-inflammatory to an anti-inflammatory, tissue-remodeling, and wound-healing proteins. These included ECM constituents Collages type1, type 3, type 4, and ECM-associated proteins Destrin, Fibulin-1, Laminin subunit β1, and Nidogen-1, among many others. There was further a distinct upregulation of anti-inflammatory and wound-healing proteins such as Plasminogen, α2M, Lactoferrin, TGF-β, Vitamin D-binding protein, SPARC, and Thrombospondin-2, all of which are known to affect the polarization of macrophages toward M2 phenotype [65,66,67,68,69,70,71]. We have reported earlier that in human myometrial cells, inflammation activates phosphorylation and nuclear translocation of NF-κB, while the BSCI significantly blocks NF-κB signaling, likely preventing the expression of pro-inflammatory mediators [29].

Notably, we found that the BSCI induced the secretion of Annexin A1 and Annexin A2 proteins. Annexins are a family of calcium-dependent phospholipid-binding proteins essential in tissue remodeling and resolution of inflammation [72,73,74,75,76,77,78,79,80]. Annexin A1 can inhibit pro-inflammatory pathways [81], reduce the recruitment of leukocytes [82], and influence macrophage polarization, promoting the shift from pro-inflammatory M1 to anti-inflammatory M2 phenotype [79,83,84,85]. Importantly, Annexin A1 works synergistically with TGF-β, a key mediator of tissue remodeling and M2 macrophage polarization [86,87]. TGF-β enhances the anti-inflammatory actions of Annexin A1, promoting the resolution of inflammation, extracellular matrix remodeling, and tissue healing.

Next, we examined whether S-CM primed by the BSCI or the BSCI itself could modulate macrophages and possibly favor an M2 phenotype. We directly treated monocytes with the BSCI in addition to S-CM, or indirectly, by first priming the myocytes with the BSCI and then treating monocytes with BSCI-primed CM. This indirect treatment through the myocyte significantly reduced M1 macrophage markers CD80 and HLA-DR, indicated by flow cytometry, while the drug had minimal direct effects on the macrophage (Supplemental Figure S2). Furthermore, mass spectrometry analysis revealed a significant decrease in pro-inflammatory M1 macrophage markers IDO, Integrin β-3, Calcium homeostatic modulator, Antigen peptide transporter 2, TNF-αR, HLA-DR1, ICAM-1, and Compliment C1q. In addition, mass spectrometry detected increased proteins associated with M2 macrophages such as CD206, CD163, Macrophage-capping protein, Annexins A1,2,4,5,6, 7,11, and associated complex-forming proteins S100A4,6,8-11. It has been reported that S100A8 and S100A9 activate inflammatory pathways, but their interaction with Annexins, notably Annexin A1 and A2, promotes a shift toward anti-inflammatory responses [88,89,90,91,92]. We further discovered that macrophages exposed to BSCI-treated media increased their Annexin A1 and A2 expression, regardless of treatment with NS-CM or S-CM. Annexin A1 can inhibit the activation of NF-κB signaling, a critical pro-inflammatory pathway that drives the production of inflammatory cytokines and the polarization of macrophages toward the M1 phenotype [93,94]. Therefore, using immunoblotting, we confirmed the ability of the BSCI-treated media to inhibit S-CM-induced NF-κB activation [29]. From these data, along with what has already been published previously [78,79,84,93,94], we propose that by suppressing NF-κB signaling, Annexin A1 drives macrophages toward the anti-inflammatory M2 phenotype, which supports tissue repair, ECM remodeling, and the resolution of inflammation [81,95].

Interestingly, gene ontology analysis of significantly differentiated proteins demonstrated the highest enrichment of the “myoblast fusion in skeletal muscle regeneration” pathway. Previously, it was shown that damaged muscle tissues secrete various proteins, including ECM components and alarmin molecules [79,96,97,98,99], which promote the reprogramming of resident macrophages to a homeostatic M2-like phenotype for muscle regeneration and wound healing. Due to this shift, the expression of Annexin A1 and A2 proteins in macrophages increased, allowing them to become more phagocytotic for the clearance of apoptotic cells and debris. We speculated that a similar phenomenon happened in uterine myocytes treated with the BSCI, where the drug induced proteins capable of transforming macrophages into a homeostatic M2-like phenotype [100,101]. Our results confirmed that NS-CM-treated macrophages are M2-like and became even more phagocytotic when exposed to BSCI-treated CM. These data indicated the M2 phenotypic modulation of macrophages exposed to BSCI-modulated myocyte secretions.

**Figure 9:**
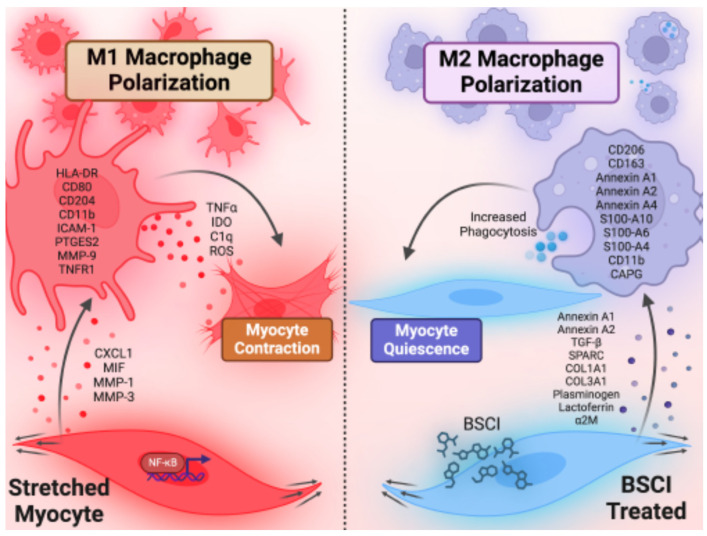
Putative model of myometrial stretch-induced macrophage activation. During late pregnancy, uterine walls are mechanically stretched by growing fetus [52]. Stretched uterine smooth muscle cells (myocytes) secrete multiple factors that induce infiltration of maternal peripheral monocytes [10,11] and promote their differentiation into resident tissue macrophages and polarization to pro-inflammatory M1 phenotype. As a feedback effect, these M1 macrophages can activate uterine myocytes to induce muscle contractions in preparation for the onset of labor [7,27,28,29]. By treating the uterine myocytes with the BSCI, we altered their secretome so they produce anti-inflammatory proteins capable of repolarizing macrophages to a homeostatic M2 phenotype which promotes myocyte quiescence.

Next, we performed a functional study using a 3-dimensional co-culture collagen gel contraction assay [27,28,29]. Our experiments showed that myocyte–macrophage co-culture of programmed M1 macrophages promoted stronger gel contraction compared to co-culture with M2 macrophages or myocytes alone. Similarly, S-CM-treated macrophages in co-culture induced a greater gel contraction compared to NS-CM-treated macrophages or control myocytes alone, while BSCI priming of myocytes prevented this. Therefore, the current results support two major conclusions (Figure 9). First, in accordance with our earlier study [10,11,17], the mechanical stretch of the uterine walls by growing fetus(es) induces myometrial secretome with pro-labor effect, resulting in leukocyte influx, differentiation/polarization of infiltrating monocytes, and tissue macrophages to a pro-inflammatory M1 phenotype. Secondly, that the BSCI modulates the myometrial secretome to one which promotes an M2-like macrophage phenotype, thereby preventing the pro-contractile, pro-inflammatory action of myometrial stretch.

While this study provides important insights into the molecular mechanisms underlying myocyte–macrophage interactions, several limitations must be acknowledged. First, the in vitro nature of our experiments may not fully capture the complexity of the in vivo uterine environment. Further validation using in vivo models is necessary to confirm the therapeutic potential of the BSCI in modulating myometrial macrophage phenotypes. Second, although our study identifies key proteins involved in macrophage polarization, additional research is needed to determine their precise functional roles and interactions within the myometrium. Future studies should explore the long-term effects of the BSCI on myometrial contractility, immune cell infiltration, and, importantly, pregnancy safety outcomes. Lastly, while our study focused on the late gestational period, investigating the role of myocyte secretome modulation earlier in pregnancy may provide further insight into the mechanisms contributing to PTB risk.

In summary, the current study offers novel insights into the mechanisms by which the myometrial-secreted factors may influence the differentiation/polarization of infiltrating monocytes and the landscape of resident macrophages during late pregnancy. Using high-throughput mass spectrometry, we characterized thousands of proteins secreted by myocytes in response to mechanical stretch, revealing a significant shift toward M1 macrophage polarization. This study builds upon prior research demonstrating the importance of macrophage in pregnancy maintenance and labor, providing novel mechanistic insights into how the myocyte secretome modulates macrophage behavior. At the same time, abundant evidence indicates that human macrophages contribute to myometrial cell activation and contraction in vitro [27,28,29]. In particular, it was demonstrated that monocytes infiltrating myometrium before labor onset participate in crosstalk that potentiate pro-inflammatory cytokine secretion [28], while macrophage-released ROS increases myocyte expression of gap junction protein CX43 and contractility [27]. We have shown the crucial role of monocytes during labor and their interaction with myometrial cells [9,17]. Moreover, recently we reported that direct physical contact between human macrophages and myometrial cells (both derived from term pregnant patients) results in increased CX43 protein expression in myocytes, while a pharmacological agent BSCI blocked Cx43-mediated myocyte–macrophage communication and contractility [29]. A key advancement of the current study is our demonstration that the BSCI can directly modulate the myometrial niche to alter macrophage polarization, shifting the inflammatory balance toward an M2-like state. By identifying the Annexin proteins as key mediators of the BSCI’s effects, this study highlights the need for further in vivo investigations to explore the therapeutic potential of the BSCI in pregnancy. Given the critical role of inflammatory pathways in the onset of PTL, interventions that can mitigate excessive inflammation while preserving normal labor processes could have profound clinical applications. The ability of the BSCI to shift macrophage polarization and modulate myometrial contractility underscores its potential as a novel therapeutic avenue for preventing PTB, addressing a major unmet medical need in obstetrics.

## Data Availability

All data generated in this study, including the raw files and quantitative data matrix of proteome, have been deposited online. Specifically, proteome datasets of the myocyte conditioned media and macrophages have been deposited to MassIVE with accession number MSV000097166, and can be accessed by using the password “Proteomics13”.

## Supplementary Materials

The following supporting information can be downloaded at https://www.mdpi.com/article/10.3390/cells14070514/s1. Figure S1: Gating strategy for differentiation and polarization of human peripheral monocytes to macrophages and image stream confirmation of fluorescence intensity distribution; Figure S2: Direct and indirect effects of BSCI on differentiation and polarization of human macrophages; Figure S3: Control M1 vs. M2 macrophage phagocytosis zymosan assay; Figure S4: Viability of collagen-embedded human myocytes–macrophage co-cultures.
