# Supplementary material for: A Broad-Spectrum Chemokine Inhibitor Drives M2 Macrophage Polarization Through Modulation of the Myometrial Secretome
Source: Cells. 2025 Mar 30;14(7):514. doi: 10.3390/cells14070514 (PMC11989072; doi:10.3390/cells14070514)
Supplement: Supplementary file 1 [file cells-14-00514-s001.zip › cells-3539193-supplementary.pdf]

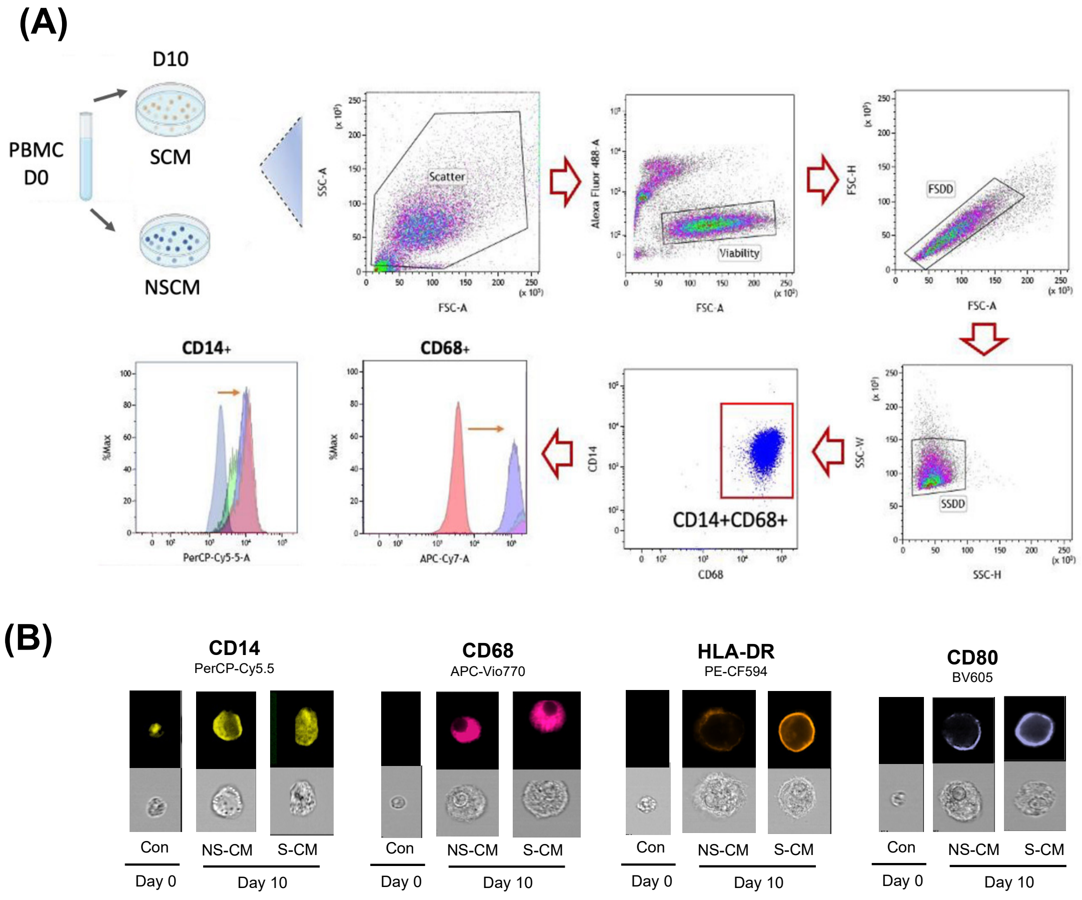

Supplemental Figure S1: Gating strategy for differentiation and polarization of human peripheral monocytes to macrophages and image stream confirmation of fluoresce intensity distribution. **(A)** Maternal peripheral blood was collected from non-laboring term pregnant women (N=6/group). Monocytes were isolated as described in methods and treated for 10 days in conditioned media (CM) secreted by mechanically stretched (S-CM) and control non-stretched (NS-CM) primary human uterine myocytes. Monocytes (Control, day 0) and differentiated/polarized macrophages (day 10) were then stained with monocyte specific marker CD14 (PerCP-Cy5.5), macrophage differentiation marker CD68 (APC-Vio770), M1 specific markers HLA-DR (PE-CF594) and CD80 (BV605) and analyzed by flow cytometry on a BD LSRFortessa™ Cell Analyzer. Data analysis was performed using BD Kaluza 2.2.1 software**. (B)** Representative images of fluorescent intensity distribution of conjugated surface markers (CD14, CD68, HLA-DR and CD80) detected on monocytes (day zero) and macrophages (day 10) using the ImageStream®X Mark II Imaging Flow Cytometer.

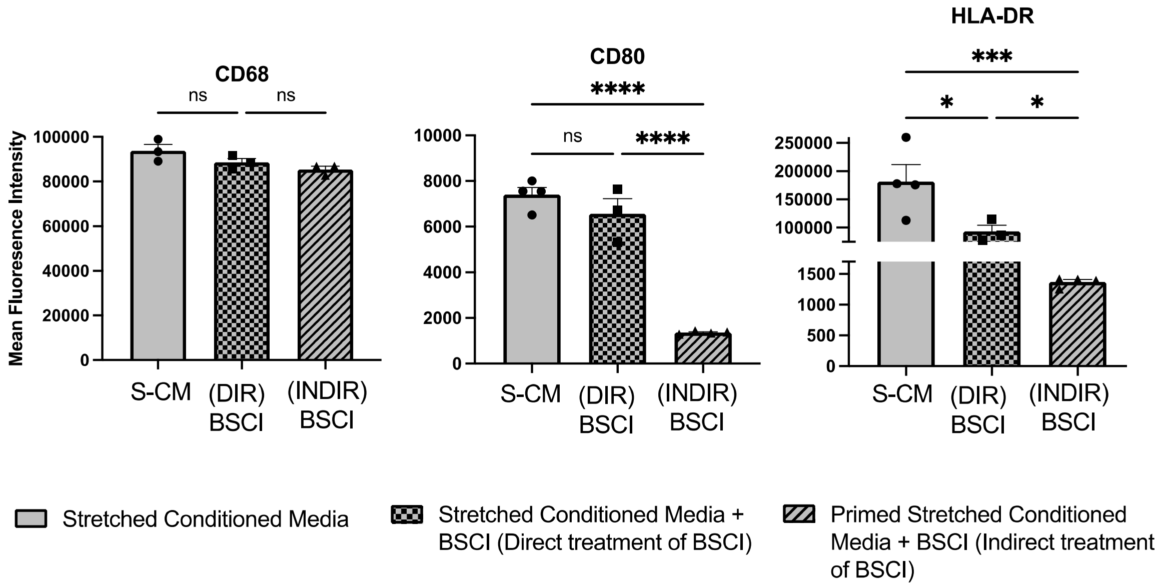

CD68
CD80
HLA-DR
Mean Fluoresence Intensity
ns
ns
****
****
***
*
*
100000
80000
60000
40000
20000
0
10000
8000
6000
4000
2000
250000
200000
150000
100000
1500
1000
500
S-CM
(DIR) BSCI
(INDIR) BSCI
Stretched Conditioned Media
Stretched Conditioned Media + BSCI (Direct treatment of BSCI)
Primed Stretched Conditioned Media + BSCI (Indirect treatment of BSCI)

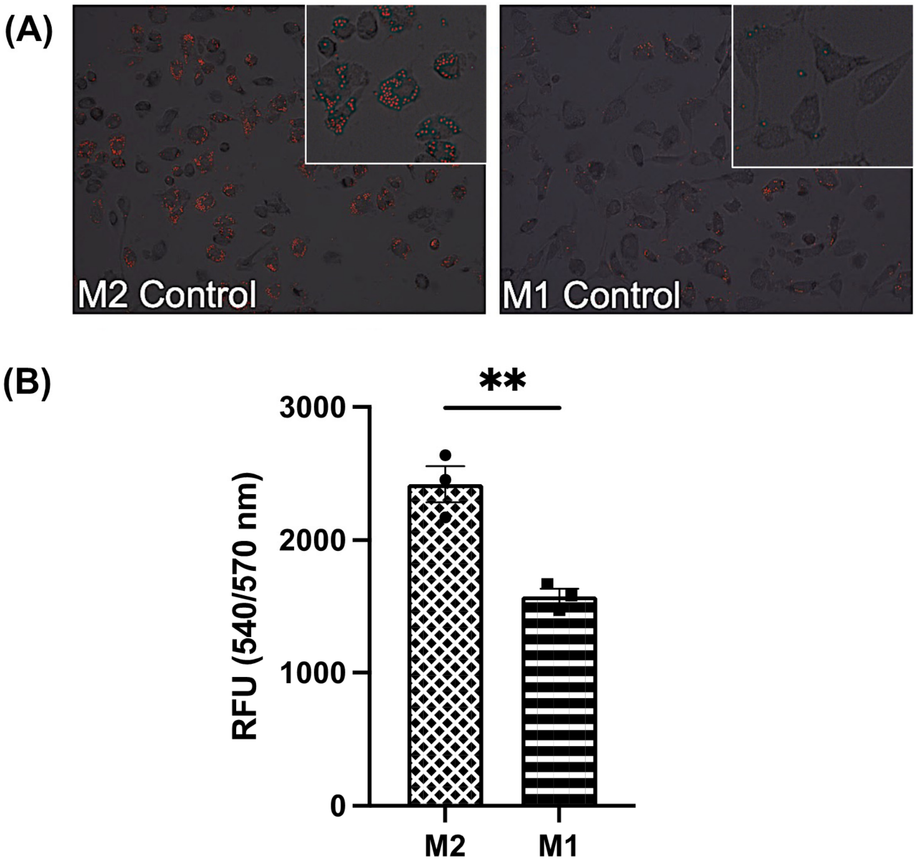

Supplemental Figure S3: Control M1 vs. M2 macrophage phagocytosis zymosan assay. Maternal peripheral blood was collected from pregnant term non-laboring women (N=3/group). For M1 polarization cells were differentiated for 6 days in media containing GM-CSF (100 ng/mL) and polarized for 2 days of LPS (100 ng/mL) and IFNγ (25 ng/mL); for M2 polarization cells were differentiated for 6 days in M-CSF (100 ng/mL), and 2 days in IL-4 (25 ng/mL), and IL-3 (25 ng/mL). After treatment, macrophages (5 x 10^4) were incubated at 37°C with 5 µl of zymosan particles for 1 hour. Plates were then evaluated by real-time confocal analysis using a Leica DM IL LED-Inverted fluorescence microscope. **(A)** Representative immunofluorescent images of zymosan particles phagocytized by macrophages. **(B)** Plates were quantified in duplicates for relative fluorescence units (RFU), and absorbance readings were conducted using µQuantTM software. The average of three macrophage lines (N=3/group) is shown. Original magnification, ×20, inlet magnification ×40. Data are presented as mean ± SD. Statistical significance was determined by one-way ANOVA followed by Dunnette's multiple comparisons test. '**' denotes statistical significance at p<0.01.

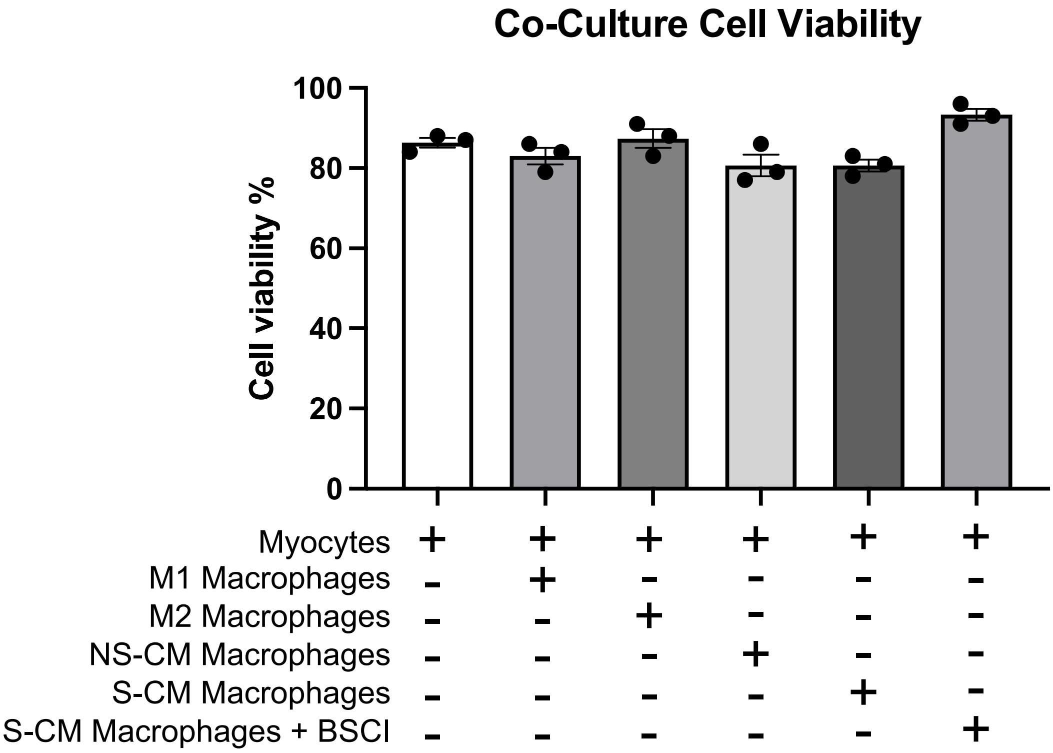

Supplemental Figure S4: Viability of collagen-embedded human myocytes-macrophage co-cultures. After experimental completion (48 hours), myocytes and macrophages from collagen gels were released via 0.1% collagenase digestion. Cells were collected by centrifugation, and viability was measured using a hemocytometer with Trypan blue viability dye. Data are presented as Mean ± SD. Statistical significance was determined through One-Way ANOVA followed by Dunnette's multiple comparisons test.
